# HPTN 071 (PopART): Rationale and design of a cluster-randomised trial of the population impact of an HIV combination prevention intervention including universal testing and treatment – a study protocol for a cluster randomised trial

**DOI:** 10.1186/1745-6215-15-57

**Published:** 2014-02-13

**Authors:** Richard Hayes, Helen Ayles, Nulda Beyers, Kalpana Sabapathy, Sian Floyd, Kwame Shanaube, Peter Bock, Sam Griffith, Ayana Moore, Deborah Watson-Jones, Christophe Fraser, Sten H Vermund, Sarah Fidler

**Affiliations:** 1 Department of Infectious Disease Epidemiology, London School of Hygiene and Tropical Medicine, Keppel Street, London WC1E 7HT, UK; 2 Department of Clinical Research, London School of Hygiene and Tropical Medicine, Keppel Street, London WC1E 7HT, UK; 3 Zambia AIDS Related TB Project, University of Zambia, Rideway Campus, Nationalist Road, Lusaka, Zambia; 4 Desmond Tutu TB Centre, Stellenbosch University, Francie van Zijl Avenue, Clinical Building, K Floor, Romm 0065, Tygerberg Campus, Western Cape 7505, South Africa; 5 FHI360, Science Facilitation Department, 2224 E NC Hwy 54, Durham, NC 27713, USA; 6 St Mary’s Campus, HIV Clinical Trials Unit, Winston Churchill Wing, London W2 1NY, UK; 7 Institute for Global Health and Department of Pediatrics, Vanderbilt University, Institute for Global Health, 2525 West End Avenue, Suite 750, Nashville, TN 32703, USA; 8 Department of Infectious Disease Epidemiology, Imperial College London, St Mary’s Campus, Norfolk Place, London W2 1PG, UK

## Abstract

**Background:**

Effective interventions to reduce HIV incidence in sub-Saharan Africa are urgently needed. Mathematical modelling and the HIV Prevention Trials Network (HPTN) 052 trial results suggest that universal HIV testing combined with immediate antiretroviral treatment (ART) should substantially reduce incidence and may eliminate HIV as a public health problem. We describe the rationale and design of a trial to evaluate this hypothesis.

**Methods/Design:**

A rigorously-designed trial of universal testing and treatment (UTT) interventions is needed because: i) it is unknown whether these interventions can be delivered to scale with adequate uptake; ii) there are many uncertainties in the models such that the population-level impact of these interventions is unknown; and ii) there are potential adverse effects including sexual risk disinhibition, HIV-related stigma, over-burdening of health systems, poor adherence, toxicity, and drug resistance.

In the HPTN 071 (PopART) trial, 21 communities in Zambia and South Africa (total population 1.2 m) will be randomly allocated to three arms. Arm A will receive the full PopART combination HIV prevention package including annual home-based HIV testing, promotion of medical male circumcision for HIV-negative men, and offer of immediate ART for those testing HIV-positive; Arm B will receive the full package except that ART initiation will follow current national guidelines; Arm C will receive standard of care. A Population Cohort of 2,500 adults will be randomly selected in each community and followed for 3 years to measure the primary outcome of HIV incidence. Based on model projections, the trial will be well-powered to detect predicted effects on HIV incidence and secondary outcomes.

**Discussion:**

Trial results, combined with modelling and cost data, will provide short-term and long-term estimates of cost-effectiveness of UTT interventions. Importantly, the three-arm design will enable assessment of how much could be achieved by optimal delivery of current policies and the costs and benefits of extending this to UTT.

**Trial registration:**

ClinicalTrials.gov NCT01900977.

## Background

The global health burden associated with HIV infection continues to grow, with an estimated 34 million people living with HIV, including 23.5 million adults and children in sub-Saharan Africa [1]. While several countries have reported reductions in HIV prevalence and incidence, prevalence remains extremely high, especially in Southern Africa which continues to experience severe, generalized epidemics with persistently high rates of HIV incidence [1].

While considerable progress has been made in expanding the coverage of antiretroviral treatment (ART), a large proportion of HIV-infected individuals who need treatment are not yet receiving it. Worldwide, it is estimated that 14.8 million adults and children are eligible for treatment under current 2012 guidelines, of which 8 million are receiving it [1]. ART is a lifelong commitment and therefore ongoing treatment costs continue to escalate as more patients require ART. In sub-Saharan Africa, an estimated 2.3 million individuals have commenced ART during the past two years, but during this same period there have been approximately 3.6 million new infections [1]. Clearly, unless the number of new infections can be steeply reduced, the number of individuals needing treatment will continue to increase and it will be increasingly difficult and costly to provide ART for all those who need it. Effective HIV prevention thus remains a pressing priority in the era of ART roll-out.

There is increasing recognition that a combination of prevention methods will be needed to bring HIV transmission under effective control in the most severely affected countries, and combination prevention programmes are being developed to meet this need [2,3]. These may involve the provision of proven prevention methods, such as male circumcision [4-7] and prevention of mother-to-child transmission (PMTCT) [8-12], a range of behavioural and biomedical interventions specially targeted at those most at risk of infection, and expanded testing and treatment for individuals found to be HIV-infected [13,14]. The potential role of earlier treatment as a preventive measure has been emphasised by an individually randomised trial showing that early treatment of HIV-infected individuals reduced transmission to their sexual partners by 96% [15].

While combination prevention strategies are based on sound epidemiological principles, they have not been adequately evaluated in the field and there are no data on their effectiveness or cost-effectiveness in reducing HIV incidence at population level [16]. This paper describes the rationale and design of a large-scale cluster-randomised trial aimed at implementing a combination prevention package including universal voluntary testing, active linkage into care, and offer of immediate ART for all those testing HIV-positive, and measuring its impact on population-level HIV incidence. This study, the HIV Prevention Trials Network (HPTN) 071 (PopART) trial, will be carried out in 21 communities in Zambia and South Africa.

### Approaches to HIV prevention in Africa

Since the principles of combination HIV prevention were formulated, there has been new interest in the potential impact of universal testing and treatment (UTT) interventions. This concept represents a paradigm shift in HIV prevention, since it focuses on identifying and intervening in HIV-infected individuals in preference to the much larger uninfected population. Mathematical modelling has indicated that if a high proportion of the population can be tested, with those found to be HIV-infected offered immediate ART, HIV infection could be reduced substantially within two years and could potentially be eliminated as a public health problem in the longer term [17-22], although the model assumptions have been questioned [23,24]. While challenging to deliver, this approach would nevertheless have major advantages in terms of simplicity and universality, potentially reducing the need for interventions targeting specific groups at high risk of infection, who are often stigmatized, as well as bringing likely clinical benefit to those infected with HIV.

Incident HIV infections necessarily result from transmission of the virus between an HIV-infected index case and an HIV-uninfected individual. HIV viral load is the key determinant of viral transmission, as demonstrated clearly in observational studies of sexual transmission among HIV-discordant couples; in those studies, no transmission was seen when the index case had a plasma viral load below 1,000 copies HIV ribonucleic acid (RNA)/mL [25,26]. By reducing plasma viral load to undetectable levels (<50 copies HIV RNA/mL), it is assumed that ART will also suppress viral burden in the genital tract to levels at which transmission is unlikely to occur [27,28], although genital shedding of HIV can sometimes occur even when plasma viraemia is suppressed [29]. While vertical HIV transmission occurs via a different route, proof of concept is provided by trials of PMTCT, which have demonstrated that HIV transmission from mother to child before, during, or after delivery is largely prevented by ART [10-12].

Of greater relevance to sexual transmission are results of the HPTN 052 trial. In this large, Phase III trial, the effects of early ART on transmission were investigated in 1,763 HIV-serodiscordant couples [15]. HPTN 052 was powered to determine the impact of immediate ART initiation for the HIV-infected partner (at CD4 counts between 350 and 550 cells/μL) on HIV transmission, compared with ART initiation at CD4 counts ≤250 cells/μL. The trial results were released early, after the early treatment arm demonstrated a 96% reduction (hazard ratio (HR): 0.04; 95% CI: 0.01–0.27; *P* <0.001) in HIV transmission to sexual partners, as well as significantly lower morbidity in HIV-infected index cases. While onset of ART at CD4 counts above 550 cells/μL was not studied in this trial, effects on HIV transmission would be expected to be similar for any given baseline viral load if viral suppression on treatment were achieved. Clinical benefits and risks of starting ART at CD4 counts above 550 cells/μL will be measured in the Strategic timing of AntiRetroviral treatment (START) trial which is scheduled to report in 2015 [30]. However, given that relatively few patients from Africa are included in this trial, the generalizability of its findings for sub-Saharan Africa may be limited [31]. Recent observational data from a large population-based cohort in South Africa have shown that the risk of HIV acquisition is inversely proportional to the local coverage of ART [32], providing additional evidence of the potential effectiveness of treatment as prevention even at lower ART initiation thresholds.

The full benefit of expanded provision of ART at population level cannot be achieved while a large proportion of the population are not aware of their HIV status, and where those diagnosed HIV-positive are not effectively linked to treatment and care services [33,34]. The UTT strategy aims to maximize the effects of ART on transmission and morbidity by promoting universal voluntary HIV testing throughout the community repeated at frequent intervals to detect new infections as they occur, effective linkage to care, and the offer of immediate onset of ART irrespective of CD4 count.

In the PopART intervention, universal testing will be promoted and provided through a house-to-house campaign delivered by specially trained community health workers known as CHiPs (community HIV care providers). There is now considerable experience from similar campaigns in different parts of sub-Saharan Africa, which have mostly achieved high rates of uptake and acceptability. A recent systematic review showed an overall average uptake of 83% and, importantly, showed similar uptake in men and women [34], whereas male clients are often under-represented at venue-based HIV testing services [35].

UTT constitutes a non-discriminatory approach whereby testing is offered community-wide irrespective of perceived risk of HIV infection and, on testing HIV-positive, everyone is offered ART irrespective of immune status. The resultant potential for normalisation of HIV could reduce stigma and have the added benefit of simplifying HIV-care services, for both patients and providers.

### UTT as part of combination prevention

Delivery of UTT is readily incorporated into a combination prevention package including other proven HIV preventive methods. Behaviour change messages have been central to most AIDS control programmes in Africa, and changes to safer sexual behaviour are assumed to have contributed to the reductions in HIV prevalence in Uganda, Zimbabwe, and other countries [36,37]. However, there is a dearth of evidence from rigorously-designed trials on what specific behavioural interventions bring about the required behavioural changes leading to a reduction in HIV incidence [38]. Similarly, while HIV counselling and testing provide the gateway to key treatment and prevention services, evidence of their effects on behaviour and HIV risk is inconclusive [39-41]. Recent findings on the population-level effects of expanded HIV testing and counselling from the HPTN 043 trial point towards a small reduction in HIV incidence in communities randomised to receive community mobilization, mobile voluntary counselling and testing, and post-test support services (HR: 0.86; 95% CI: 0.73–1.02; *P* = 0.08), compared to communities with standard voluntary counselling and testing services alone [42].

In contrast, stronger evidence of effectiveness is available for some biomedical interventions. Male circumcision was shown to reduce HIV incidence by around 60% in three trials in Kenya, South Africa, and Uganda [5-7]. Safe services for medical male circumcision have been recommended for wide-scale roll-out by the World Health Organization (WHO) and the United Nations Programme on HIV/AIDS (UNAIDS), although progress in implementation in many countries has been slow [43,44]. HIV transmission is known to be facilitated by other sexually transmitted infections (STIs) [45]. One trial in Tanzania showed that improved STI treatment services reduced HIV incidence in the general population, although other trials of a variety of STI interventions in different epidemiological settings have failed to show an impact on HIV incidence [46]. PMTCT services have been shown to have a major impact on the rate of vertical transmission from HIV-positive pregnant women to their infants [10-12].

### Why are trials of UTT needed?

There is a very strong biological and epidemiological rationale for the potential efficacy of universal HIV testing combined with immediate onset of ART. Many countries and agencies are promoting wider delivery and uptake of HIV testing in the community with the aim of ensuring that all adults know their HIV status [47-50]. There is increasing recognition of the potential value of starting ART earlier, both for individual clinical benefit and to prevent onward transmission, and international and national treatment guidelines have moved steadily towards higher CD4 thresholds [51-54]. In the USA, many clinicians are already prescribing ART for many of their HIV-positive patients at CD4 counts well in excess of 500 cells/μL. Some mathematical models have demonstrated that if UTT can be delivered with high coverage, HIV incidence could be reduced substantially [18-20,22].

Some have argued that, given this array of evidence and the pressing public health need, UTT interventions should be implemented immediately without the need for supporting trials. We argue that rigorously designed trials are needed for several cogent reasons [16].

First, it is not known whether UTT interventions can be implemented on a large-scale in resource-poor settings and whether they can achieve high uptake and acceptability. To achieve high impact, such interventions will need to achieve high coverage of HIV testing, which needs to be repeated at frequent intervals. Those diagnosed HIV-positive need to be effectively and rapidly linked to HIV treatment and care services and to be started on ART without delay. Patients started on ART also need to maintain high levels of adherence in order to ensure effective viral suppression over a prolonged time period. Interventions may fail at one or more of these three critical stages.

Second, while findings from mathematical models are encouraging, such models by necessity make numerous assumptions, some of which are insufficiently supported by empirical data. The initial models of UTT, showing substantial effect sizes, have been criticised for over-optimistic assumptions. Other modelling groups, using different approaches and assumptions, have produced a wide range of projections, although most of these agree in suggesting substantial impact [22].

Third, while the results of the HPTN 052 study show convincing evidence of effects on transmission in individual partnerships in a clinical trial setting, the population level impact of UTT is unknown [15]. It could be much smaller because routinely provided interventions may not achieve sufficiently high levels of uptake and adherence. Conversely, there may be additional indirect effects if there is high coverage of the intervention at population level. The net benefits are very difficult to assess convincingly without an empirical study.

Fourth, and importantly, there are many potential adverse effects of large-scale UTT interventions. They will require effective health educational messages that alter the perception that ART is reserved for those who are sick and emphasise that ART is now intended for all HIV-infected individuals, the majority of whom will not have any indication of disease. Where this messaging is not achieved, ambivalence to ART could be anticipated. This could be accompanied by low levels of ART adherence, leading to the development of drug resistance. Associated with this risk, there is also the potential for increased transmission of resistant viruses as well as the need for more expensive or toxic second-line regimens. Uncertainties remain around the risks and benefits of prescribing life-long treatment to healthy individuals with high CD4 counts. The widespread provision of ART may lead to sexual risk disinhibition among either treated individuals or the general population, resulting in higher incidence of HIV and offsetting the protective effects of the intervention. While the universal approach to testing and care enshrined in UTT is designed to reduce HIV-related stigma, possible harm through involuntary disclosure of HIV status and other social harms cannot be ruled out. Finally, health services in many endemic countries are already struggling under the burden of providing care with the current restricted approach to ART delivery. There is genuine concern that more than doubling the number of patients on ART may severely over-stretch clinic resources, leading to a decline in the quality of care for HIV and other health conditions.

A rigorously designed trial will provide clear evidence of the balance of risks and benefits, and reliable estimates of the effectiveness and cost-effectiveness of the UTT strategy in reducing HIV incidence at population level. Such evidence would likely be of great value to policy makers in the study countries, other parts of sub-Saharan Africa and globally.

## Design

### Overall design of HPTN 071 (PopART) trial

HPTN 071 (PopART) will measure the impact of the PopART combination prevention intervention package on HIV incidence at population level by means of a cluster-randomised trial in Zambia and South Africa. The trial will be carried out in 21 study clusters – 12 in Zambia and 9 in South Africa. A cluster will be defined as the catchment population of a government primary health care facility which provides ART services to the population in the community.

There will be three study arms with 7 clusters in each arm:

Arm A: Clusters in this arm will receive the full PopART combination prevention package including immediate offer of ART, irrespective of CD4 count, for all adults diagnosed with HIV infection.

Arm B: Clusters in this arm will receive the full PopART package except that ART will be provided according to current national treatment guidelines (i.e., current CD4 threshold of 350 cells/μL in both countries).

Arm C: Clusters in this arm will continue to receive the current standard of care provided in these communities.

**Figure 1:**
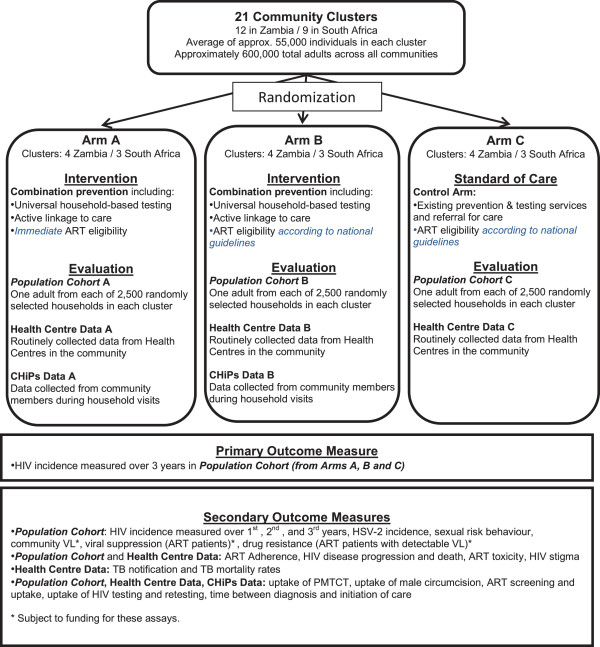
Summary of trial design.

The primary outcome, HIV incidence, will be measured in a randomly selected Population Cohort in all 21 clusters. The overall study design is summarised in Figure 1. The full study protocol is available online [55], but key components are summarised below.

### Components of intervention in Arms A and B

The full PopART combination prevention package will include the following components:

• Home-based voluntary HIV testing and counselling offered to all residents: home-based visits will be repeated at annual intervals, and all those without a prior HIV-positive diagnosis will be encouraged to re-test each year. The home-based testing service will be carried out by the CHiP team that will also support other components of the package.

• Linkage to care: those diagnosed HIV-positive will be referred to the local primary health facility for HIV treatment and care. The CHiPs will be responsible for ensuring effective linkage to care, and will follow-up HIV positive individuals who have not presented at the clinic for care within a defined period.

• Male circumcision: the CHiPs will also encourage men who test HIV-negative and who are uncircumcised to present to locally available services for voluntary medical male circumcision.

• Condom promotion: during the household visits, the CHiP team will provide behavioural risk reduction counselling and will offer a supply of condoms. Free condoms will also be available at the local health facilities and in the communities.

• Screening for symptoms suggestive of tuberculosis (TB) and sexually transmitted infections, with referral to the local health care facility for appropriate further management as necessary.

• PMTCT: the CHiP team will identify any women who are pregnant and encourage them to present for antenatal care through the local health services. Pregnant women who are diagnosed HIV-positive will be encouraged to access PMTCT services. In Arm A, all HIV-positive women will be offered immediate ART irrespective of their CD4 status.

• HIV treatment and care: all HIV-positive individuals will be encouraged to register for HIV treatment and care at the local primary health care facility. In Arm A, ART will be offered immediately to all HIV-infected adults, irrespective of CD4 count, while in Arm B, ART will be initiated according to national guidelines. Prophylaxis against TB and other opportunistic infections will be provided according to national guidelines. Clinical follow-up and monitoring will be provided according to standard clinic procedures, with additional adherence support from the CHiP teams or other community health workers.

Aside from the implementation of immediate ART in Arm A, any changes to national treatment guidelines over the duration of the trial will be adhered to in all communities.

Arm C clusters will continue to receive the current standard of care, and the CHiP services described above will not be delivered in these communities.

### CHiP teams

As described above, home-based testing and referral for services will be carried out by a cadre of staff designated as community HIV care providers (CHiPs). A CHiP is a member of the community, appointed to provide a package of basic services at the household level. CHiPs will work in pairs, in gender-balanced teams, and will have been recruited with several requirements in mind. They will have to be at least 18 years of age; have adequate reading and writing skills; be able to speak, read, and write in English and be conversant with local languages; be able to record data using an electronic data capture device; be trained and licensed in HIV counselling (including child, couple, family counselling) and testing (Zambia) or receive training after employment (South Africa); be willing to undertake training according to national or study requirements for community health worker staff (training in psychosocial counselling, HIV counselling and testing, adherence counselling, good clinical practice, and basic knowledge of HIV and TB prevention, treatment, and care); and be conversant with the local geography. They will also have to be physically able and willing to walk long distances; be able to maintain client confidentiality; and preferably reside in the community of the PopART intervention. Previous experience as a community health care worker and prior experience in basic counselling, HIV psychosocial counselling and testing, and adherence counselling will be desirable. People living openly with HIV in the community and other HIV advocates will be welcomed.

The ratio of CHiPs to household members varies by community depending on the density of households and other local factors. Over 700 CHiPs will be deployed across the 14 communities of Arms A and B in both countries.

### Study population

This study will be carried out in areas of Zambia and South Africa that are known to have high HIV prevalence and incidence and are continuing to experience severe generalized HIV epidemics, with prevalence levels of 15% to 20% in many communities. National estimates of HIV prevalence in adults aged 15 to 49 are 13.5% for Zambia and 17.8% for South Africa, and incidence estimates are 1.06% [56] and 1.49% [57], respectively.

**Figure 2:**
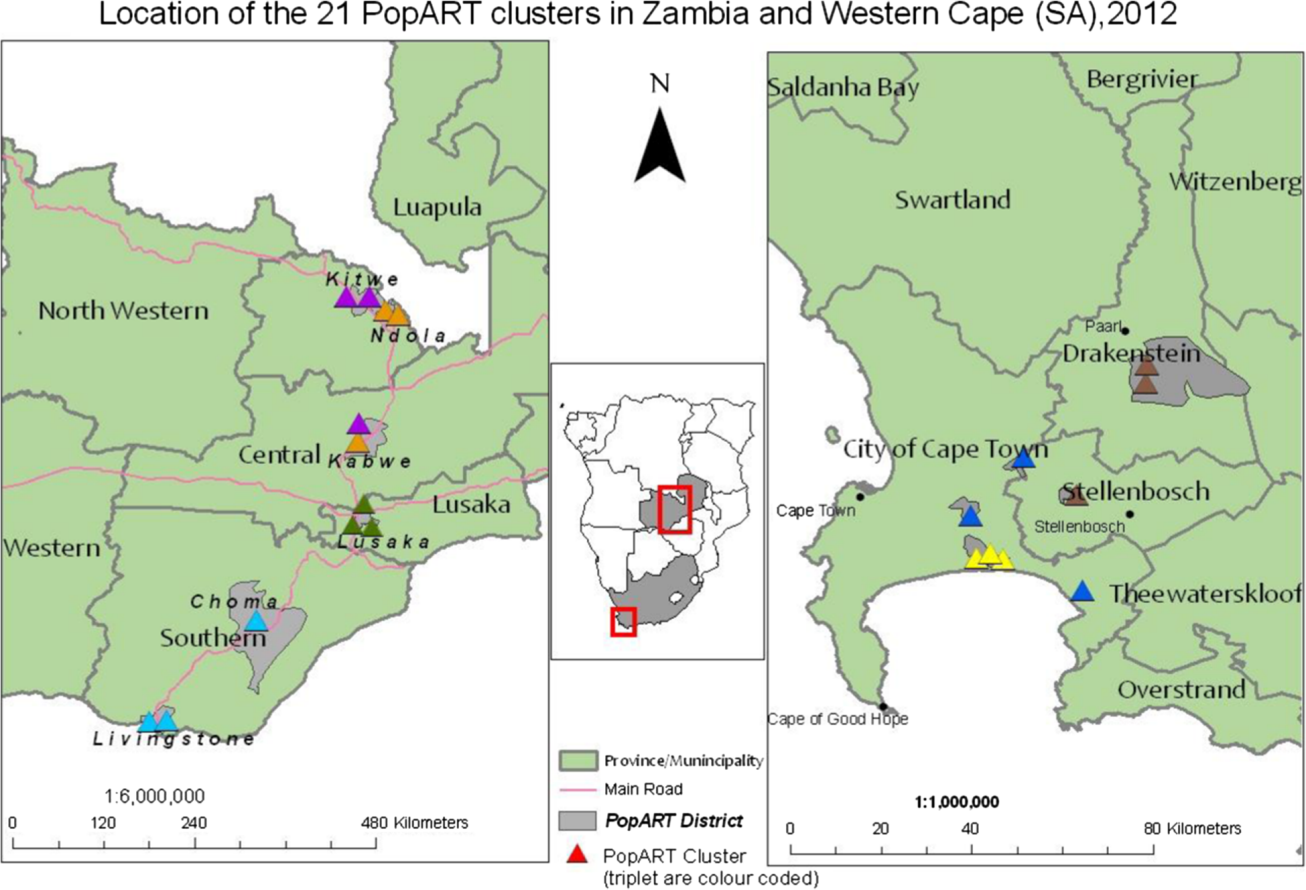
Map showing location of study communities.

The 21 study communities are shown in Figure 2. The Zambia South Africa TB and AIDS Reduction (ZAMSTAR) cluster-randomised trial [58] was previously carried out in the same or nearby communities. The HPTN 071 (PopART) trial will benefit from the strong community and stakeholder relations built up during the ZAMSTAR trial, as well as epidemiological data on HIV and TB that have been used to aid the design of the current trial.

Additional considerations that informed the selection of the 21 communities for the current study were:

• They are as far as possible geographically distinct.

• No other major HIV prevention studies are planned or ongoing.

• There is an adequate population size to minimize the effects of contamination on outcome measurements due to contact with other communities or residents of other communities.

• Community willingness to be involved in the current trial.

Key characteristics of the study communities are summarised in Table 1. For 14 trial communities, these data were available from the 2010 TB prevalence survey of the ZAMSTAR trial, while for seven Western Cape communities estimates were obtained from routine data, or data from neighbouring ZAMSTAR trial communities. Total population size (all ages) ranges from 21,386 to 166,251 with an average of 57,828 (66,864 in Zambia, 45,780 in South Africa). The three largest communities, with populations exceeding 120,000, are all suburbs of the capital city (Lusaka) in Zambia. The total population of all 21 communities is approximately 1.2 million, based on 2001 and 2011 census data for Zambian and Western Cape communities, respectively, and this will include approximately 800,000 in the 14 intervention communities.

**Table 1:** Characteristics of study communities

**Country**	**Triplet**	**Community number**	**Adult HIV prevalence (%)**^**1**^	**HIV-infected on ART (%)**^**2**^	**Population size**^**3**^	**Men circumcised (%)**
Zambia	1	1	16	23	42,898	16
	1	2	13	29	33,297	17
	1	3	17	15	38,081	7
	2	4	19	30	60,222	17
	2	5	17	18	45,234	12
	2	6	19	32	34,623	8
	3	7	16	13	129,221	8
	3	8	15	22	166,251	8
	3	9	16	25	124,284	19
	4	10	25	24	31,629	14
	4	11	18	27	55,011	21
	4	12	16	38	41,615	14
South Africa	5	13	19	35	34,096	87
	5	14	19	35	21,386	Data unavailable
	5	15	19	35	38,059	Data unavailable
	6	16	15	37	72,544	Data unavailable
	6	17	18	28	37,084	Data unavailable
	6	18	14	36	44,821	53
	7	19	11	25	36,009	Data unavailable
	7	20	11	25	82,953	Data unavailable
	7	21	12	18	45,067	Data unavailable

^1^Estimated from ZAMSTAR 2010 TB/HIV prevalence survey, for all Zambian communities, with age standardisation to the age structure of prevalence survey participants and assuming 50% of the adult population are men. For Western Cape communities, source of HIV prevalence data varies by triplet. For Triplet 5, community 13 was included in the ZAMSTAR trial and the ZAMSTAR 2010 TB/HIV prevalence survey data are used, as for Zambia. HIV prevalence is then assumed to be the same in communities 14 and 15. For communities 16, 17, 19, 20, and 21, sub-district level data on antenatal clinic (ANC) prevalence were used, with the assumption that adult HIV prevalence is 80% of the ANC prevalence value. Community 18 was included in the ZAMSTAR trial and the ZAMSTAR 2010 TB/HIV prevalence survey data are used.

^2^Estimated from ZAMSTAR 2010 TB/HIV prevalence survey data, for all Zambian communities. The number of HIV-positive adults among prevalence survey participants was estimated, separately for men and women, as the age-standardised HIV prevalence multiplied by the number of survey participants. The proportion of HIV-positive individuals on ART was then calculated as (number self-reported on ART)/(estimated number of HIV-positive survey participants), and assuming that 50% of the adult population are men. For Western Cape communities, data were used from October 2012 on (a) the number of individuals aged >15 years old on ART – measured either at community or sub-district level, (b) population size among individuals >15 years old – measured using census data either at community or sub-district level, and (c) HIV prevalence estimates. The number of HIV-positive individuals aged >15 years old was estimated as HIV prevalence × community (or sub-district) population size. The proportion of HIV-positive individuals on ART was then calculated as (number of individuals >15 years old on ART)/(estimated number of HIV-positive individuals aged >15 years old).

^3^Population size – for Zambia, based on 2001 census data; for Western Cape, based on 2011 census data.

Adult HIV prevalence ranges from about 11% to 25%, with an average of around 15% to 17% in both countries. The proportion of HIV-infected adults on ART is more variable and ranges from 13% to 38%, averaging around 25% in Zambia in 2012 and 30% in South Africa in 2012. The prevalence of male circumcision is much higher in the communities in South Africa (>50%) than in Zambia (8–21%) as shown in Table 1.

### Randomisation

Randomisation was carried out at public ceremonies held simultaneously in Zambia and South Africa in February 2013. The 21 study communities were first grouped into 7 matched triplets, 4 in Zambia and 3 in South Africa, based on geographical proximity, implementing partners for HIV services and the best available estimates of adult HIV prevalence. The matched design was used with the aim of minimizing between-community variance in baseline HIV incidence, which is assumed to be correlated with baseline HIV prevalence. With a requirement to match on geographical area, implementing partners for HIV service provision, and HIV prevalence, it was not possible to match simultaneously on estimated uptake of ART among HIV-positive individuals or cluster size. It is acknowledged that this may increase somewhat the variability in effect size across triplets. However, restricted randomisation will help to ensure good balance in average ART uptake and cluster size across trial arms A, B, and C, as explained below.

After dividing the 21 clusters into 7 matched triplets (Figure 2), allocation to the three study arms was carried out using a process of restricted randomisation. This procedure was used to ensure overall balance across study arms on cluster size, current ART uptake, and HIV prevalence. There were (3!)^7^ = 279,936 possible ways of allocating the clusters to the three study arms within matched triplets. These allocations were evaluated against balance criteria to determine a restricted list of allocations that achieve adequate balance on the three variables defined above. The final allocation was selected randomly from this restricted list of balanced allocations. These methods were based on randomisation procedures used successfully in the ZAMSTAR trial [58].

### Evaluation of impact

While the interventions will be delivered to the total population of each intervention community (average population size 57,828), the primary outcome of HIV incidence and a range of secondary outcomes and process measures will be obtained through a *Population Cohort*. This cohort will consist of a random sample of 2,500 adults in each community (52,500 individuals in total) who will be surveyed at baseline and then again after 12, 24, and 36 months. The baseline survey will take place at roughly the same time as intervention delivery commences in the same community. The impact of the interventions will be measured by comparison of study outcomes across the three study arms in the *Population Cohort* during the 36 month follow-up period, rather than through before-and-after comparisons.

Prior to the commencement of the trial, a household census will be completed in all trial communities, providing a map and listing of all households, and including a count of the number of adults and children in each household. The household listing will be used to select a random sample of households which will be visited over a 6 to 9 month period. During the visit to a selected household, all household members will be enumerated and a random number generator will be used to select one adult resident aged 18 to 44 years. Following informed consent, the randomly selected adult will be invited to join the *Population Cohort* if they satisfy eligibility criteria, in particular with respect to residency in the community and an intention to remain resident for the next 3 years.

Only one adult will be randomly selected for the *Population Cohort* from each randomly selected household. This is to avoid the distortion of the trial results which might occur if whole households or several members of a household were to be evaluated, since this would in itself constitute a mass testing and counselling intervention. In particular, it is likely that many of the HIV-infected spouses and regular partners of *Population Cohort* members would be diagnosed and treated, thus reducing HIV transmission in all three study arms. The statistical analysis will take into account the different sampling probabilities resulting from the selection of one individual irrespective of household size. The eligible age-range of 18 to 44 years was chosen because adults aged 18 years and over are able to provide their own consent to participate in the research, because a high proportion of infections are expected to occur in the 18 to 44 group based on past data from these populations, and because HIV incidence among adults aged 45 years or older is much lower than among younger adults.

On enrolment and during each annual follow-up of the *Population Cohort*, participants will be asked to complete an interviewer-administered questionnaire covering socio-demographic information and a wide range of behavioural and HIV-related variables. At the end of the interview, blood specimens will be taken and transported to study laboratories for testing. All *Population Cohort* participants will also be offered on-the-spot voluntary counselling and testing using rapid HIV test kits. All HIV-infected individuals (those testing positive on the rapid test as well as those who are already aware of their positive status) will be referred to a health centre for further management.

Additional secondary outcomes and process measures will be measured in the *Population Cohort* or through data collected routinely by the health services or CHiP teams, as described below.

### Study outcomes

The primary outcome will be HIV incidence during 36 months of follow-up measured through prospective follow-up of the Population Cohort.

The wide range of secondary outcomes include:

• HIV incidence over each of the first, second, and third years of follow-up in the *Population Cohort* to track if the impact on population-level incidence increases over time.

• Community viral load* will be estimated by carrying out HIV viral load testing of a random sample of 75 HIV-infected members of the *Population Cohort* in each community at the baseline, 12-month and 36-month surveys, and all HIV-infected members (estimated as about 300 per community) at the 24-month survey. Various measures of community viral load, reflecting average viral load across the population, have been proposed as potential surrogate markers of the population-level success of interventions involving ART for prevention [59,60].

• ART adherence and viral suppression*: HIV viral loads of members of the *Population Cohort* who commence ART after the start of the trial will be measured at the 24 month survey (see above). Viral load testing of all HIV-infected participants in this survey will provide adequate data on viral suppression in patients who have been on ART for periods of 12–24 months. Self-reported adherence in these patients will also be analysed. Further data on loss to follow-up, treatment adherence, and viral suppression will also be obtained from health service data.

• ART drug resistance* will be measured at 24 months in members of the *Population Cohort* who are not virally suppressed, followed by back-testing of their baseline and 12-month specimens, to assess the incidence of acquired resistance among those commencing ART after the start of the trial. ART drug resistance will also be measured among members of the Population Cohort with incident infection during follow-up to assess the incidence of transmitted resistance.

• HIV disease progression, retention in care, death, and ART toxicity: these outcomes will be measured both in members of the Population Cohort and in the wider population using routine health service data.

• Sexual risk behaviour will be measured in the Population Cohort at each survey and used to assess whether there is behavioural disinhibition related to the intervention. This analysis will be supported by data collected in the Population Cohort on Herpes simplex virus, type 2 incidence, which has been shown to be a biomarker for sexual risk behaviour especially among young people [61-63].

• HIV-related stigma will be assessed through analysis of self-reported data in the Population Cohort as well as through qualitative research (see below).

• Case notification rates of TB and TB mortality among these cases will be assessed through routine health service data. TB case rates may be influenced by higher levels of ART in the intervention arms and by active case finding by the CHiP teams.

* Funding for these investigations is pending.

### Process measures

Several process measures will be recorded in all three study arms to evaluate the implementation and delivery of the PopART interventions. These measures evaluate processes that are intermediary between the provision of the intervention and achievement of the primary outcome, and will therefore be of value in understanding the results of the trial as well as learning lessons for future implementation of the intervention package.

Each of the following measures will be estimated using a combination of data from the Population Cohort, the CHiP teams, and routine health centre data:

• Uptake of HIV testing and re-testing.

• Time between HIV diagnosis and initiation of care.

• Uptake of ART screening and initiation of treatment.

• Uptake of services for PMTCT.

• Uptake of medical male circumcision.

### Case–control studies

Three nested case–control studies will be carried out to examine factors related to uptake of different components of the PopART intervention.

The first case–control study will examine uptake of HIV testing during the first round of home-based testing. Cases will be those who refuse testing by the CHiP team and controls will be those who accept testing, excluding those already known to be HIV-infected. A random sample of 400 cases and 400 controls will be chosen from the study communities in Arms A and B, and standardized questionnaires will be used to collect data on sexual and health seeking behaviour, previous HIV testing, as well as stigma and psycho-social questions. Cases and controls will also have separate sections in the questionnaire, to explore reasons for not testing and motivation to test, respectively.

The second case–control study will examine linkage to care and initiation of ART and will be carried out in Arm A only. Cases and controls will be selected from those identified as HIV-positive by the CHiP teams and who are not already taking ART. Cases will be those who have not presented for care and initiated ART within 3 months, and controls will be those who do initiate ART within this time-frame. A random sample of 400 cases and 400 controls will be chosen from the study communities in Arm A.

Finally, the third case–control study will examine uptake of HIV testing during the second round of home-based testing in the second year of the intervention, using similar methods to those for the first case–control study.

### Qualitative research

Qualitative studies will be conducted alongside the trial in both Zambia and South Africa to provide important contextual data and a more in-depth exploration of community response to the PopART intervention. These studies will include:

• Preliminary formative research in all 21 study communities using participatory rapid appraisal tools. This research was carried out to rapidly collect background information on salient features of each community, including geographical, cultural, socio-economic, and health-related characteristics. Information was also collected on the HIV landscape of each community, including attitudes and perceptions towards HIV, existing provision of preventive and treatment services, and key stakeholders. Findings from these surveys have been used to prepare a descriptive account of each community which can be used by the study team and implementing partners to inform the delivery of the intervention and research activities.

• Evaluation of the acceptability of the intervention: research will be carried out in study communities in Arms A and B at intervals during the trial to examine the response of the community over time to the different components of the intervention. A mix of methods will be used, including fieldworker structured diaries, in-depth interviews, focus group discussions, structured observation, and participatory rapid appraisal tools. Data will be collected from local stakeholders, CHiP teams, and community members from different age and gender groups. Research on the process of community engagement, and on how to ensure that research is carried out in accordance with ethical guidelines, will also be embedded in this work.

• Longitudinal study: a representative sample of cases and controls from the first case–control study will be recruited to this study and will be invited to give in-depth interviews at three-monthly intervals until the end of the trial. This study in Arms A and B will complement the findings of the case–control studies, and will document the longitudinal trajectory of individual behaviour in relation to uptake of HIV services.

• An ethnographic study, conducted in selected communities in Arms A and C, will provide contextual understanding of how communities experience the roll-out of the PopART combination prevention interventions. This research will also examine a range of related issues including HIV-related stigma, the role of welfare and food security, sexual risk disinhibition, alcohol and drug use, male circumcision, and the influence of different stakeholders and social networks.

### Mathematical modelling and economic evaluation

The design of the trial is benefiting from support from the mathematical modelling team. The initial modelling work provided estimates of projected impact over different time-scales and informed the sample size calculations for the trial, as discussed below. During the conduct of the trial, a more sophisticated stochastic model of HIV transmission will be developed and fitted to data from the trial, routine data, and published sources to address several objectives.

• To help interpret the results of the trial: Process data showing the extent of uptake of the intervention compared with similar data from the control arm will be used to obtain model projections of expected impact under these conditions. By examining projected impact under the conditions prevailing in Zambia and South Africa, and in different trial communities, we will be able to examine whether the level of impact and variations in impact are in accordance with expectations.

• To project longer-term impact: The trial will measure the impact of PopART interventions over a 3 year period. Models fitted to the impact seen during the trial will be used to project the likely impact over longer time periods.

• To explore likely impact in different settings: If the trial demonstrates impact, it is likely that similar interventions will be implemented in a wide range of settings. Therefore, the model will be used to explore how impact would be expected to vary depending on epidemiological, demographic, and other characteristics of populations, and thus to project likely impact in a range of settings.

• To explore the likely impact of alternative intervention packages: Our study design will provide empirical data on the impact of the specific packages of preventive interventions incorporated in the PopART programme. However, the model can be used to explore the effect of adding or removing components. For example, we can project the impact of an intervention in which male circumcision is not promoted, or where the threshold for starting ART is set at different levels.

To optimise the value of the trial for health policy development, it is important that data are collected on cost and cost-effectiveness as well as effectiveness. The economic evaluation will assess the incremental health benefits of the PopART intervention in relation to its incremental costs, which will be estimated by comparing health services utilisation and associated costs between the three study arms. The main focus will be on costs to the health services including equipment, materials, and personnel. The economic evaluation will rely mainly on data on health status and health service usage collected from the Population Cohort, together with health facility data.

Benefits will be assessed in terms of lifetime change in quality adjusted life years (QALYs) and/or disability adjusted life years (DALYs). By combining cost data with impact estimates from the trial and model projections, it will be possible to derive estimates of cost per QALY and/or DALY using different time horizons. We will also be able to explore the projected cost-effectiveness of alternative prevention packages.

### Statistical considerations

#### Intervention targets

The trial design has been guided by the results of mathematical modelling. The preliminary PopART model is described in a related paper [64] and was fitted to routine HIV data from Zambia and South Africa, as well as data from the ZAMSTAR trial which was carried out in the same study areas. The model was used to explore the projected impact of the PopART interventions at different levels of coverage, and this informed the choice of intervention targets.

Based on this work, Table 2 shows two sets of intervention targets, a central target and an optimistic target, for four key coverage indices. These include a target of 70% to 75% annual coverage of the test and treat campaign, which means that 70% to 75% of HIV-positive individuals not already on ART would be diagnosed, linked to care, and started on ART within 3 months. Other targets are to keep the annual rate of treatment failure or drop-out below 10% among those on ART; effectiveness of 90% to 95% of ART in blocking HIV transmission to sexual partners; and 50% uptake of male circumcision among HIV-negative men who are not already circumcised. Based on these intervention targets, Table 2 shows the projected impact on HIV incidence in Arms A and B compared with Arm C in each country and over different time periods.

**Table 2:** Parameter values assumed for the model of the impact of the intervention for central and optimistic target scenarios, and projected impact on HIV incidence in Arms A and B compared with Arm C, assuming intervention roll-out over a 6-month time period

**Parameter**		**Central target**		**Optimistic target**	
Annual coverage of test and treat campaign		70%		75%	
Treatment failure & drop-out rate, per year		10%		10%	
Effectiveness of ART in blocking transmission		90%		95%	
Take up of male circumcision when offered		50%		50%	
		Arm A	Arm B	Arm A	Arm B
Zambia	Impact on cumulative incidence (3 years)	61%	25%	63%	27%
	Impact on cumulative incidence (first 2 years)	58%	24%	61%	25%
	Impact on HIV incidence during Year 1	51%	20%	54%	21%
	Impact on HIV incidence during Year 2	65%	27%	67%	28%
	Impact on HIV incidence during Year 3	67%	29%	68%	30%
South Africa	Impact on cumulative incidence (3 years)	62%	26%	64%	27%
	Impact on cumulative incidence (first 2 years)	59%	25%	61%	26%
	Impact on HIV incidence during Year 1	52%	22%	55%	23%
	Impact on HIV incidence during Year 2	65%	28%	67%	29%
	Impact on HIV incidence during Year 3	68%	29%	69%	30%

The projections assume that the intervention is rolled out over a 6 month period during each annual round. In Zambia, for example, if the central targets are achieved for each aspect of the intervention, then, over 3 years of follow-up, HIV incidence in Arm A is expected to be 61% lower than HIV incidence in Arm C, whereas it will be 25% lower in Arm B than in Arm C. For example, if HIV incidence in Arm C is 1 per 100 person-years – it will be reduced to 0.39 per 100 person-years in Arm A. If, on the other hand, the intervention achieves the optimistic targets when rolled out, then HIV incidence will be reduced by 63% in Arm A and 27% in Arm B when compared with Arm C. These projections vary little according to the assumed level of HIV incidence in Arm C, and so are not very sensitive to background trends in incidence. The projections indicate that the predicted impact over 3 years is 61% to 64% in Arm A and 25% to 27% in Arm B. Impact is substantially higher in Years 2 and 3, as expected. As a sensitivity analysis, assuming roll-out takes 12 rather than 6 months, projected impact over three years is 58% to 61% for Arm A and 24% to 26% for Arm B (data not shown – see related paper for details) [64].

The coverage of the test and treat intervention is essentially the product of two parameters – the proportion of the population that accepts HIV testing and the proportion of those testing HIV positive who link to care and initiate treatment when eligible. The sensitivity of the projected impact on the primary outcome (reduction in HIV incidence over 3 years) to variation in these key uptake measures has been assessed (with low and high values of 40% to 95% for each of parameter); a strong linear relationship was found [64]. It will therefore be possible to update our projections once process data on uptake are available.

#### Sample size

The primary outcome will be the incidence of HIV infection measured among initially HIV-uninfected members of the *Population Cohort* during the follow-up period of 36 months. Based on national estimates of HIV incidence and on HIV prevalence in the chosen study areas, it is expected that HIV incidence in the control arm will be in the range 1.0 to 1.5/100 py. With a matched study design, and based on estimates of between-community variation from the ZAMSTAR trial in the same study areas, it is expected that the between-community coefficient of variation will be in the range 0.15 to 0.20. Seven communities were chosen per study arm and a *Population Cohort* of 2,500 adults per community to attain adequate power to detect a difference in incidence between Arms A and C (reflecting the full impact of the intervention), as well as the difference in intervention effect between Arms A and B (reflecting the additional effect of immediate HIV treatment compared with current national guidelines) [65,66].

Table 3 shows that the study will be very well powered to detect an effect of 35% or larger in Arm A or Arm B compared with Arm C, and moderately well powered to detect an effect of 30% under favourable assumptions. For the direct comparison of Arms A and B, Table 4 shows that the study will be well powered to detect a difference between effects of 60% and 30%, 55% and 25%, and 50% and 20%. Tables 3 and 4 allow for a baseline HIV prevalence of 15% and assume losses to follow-up of 20% over two years, and 25% over three years.

**Table 3:** **Power for comparison of HIV incidence in Arm A or B with Arm C, with 7 communities per arm and *Population Cohort *** **of 2,500 adults per community (assuming that on average 2,125 (85%) will be HIV-uninfected at baseline and that loss to follow-up will be 20% after 2 years and 25% after 3 years) with 5,206 person-years per community over 36 months**

**HIV incidence rate/100 py (control arm)**	**Between-cluster coefficient of variation (k)**	**Effectiveness (%)**	**Power (%)**
1.0	0.15	25%	57%
1.0	0.15	30%	74%
1.0	0.15	35%	87%
1.0	0.15	40%	95%
1.0	0.15	45%	99%
1.0	0.15	50%	100%
1.0	0.15	55%	100%
1.0	0.15	60%	100%
1.0	0.15	65%	100%
1.0	0.20	25%	44%
1.0	0.20	30%	60%
1.0	0.20	35%	75%
1.0	0.20	40%	87%
1.0	0.20	45%	94%
1.0	0.20	50%	98%
1.0	0.20	55%	99%
1.0	0.20	60%	100%
1.0	0.20	65%	100%
1.5	0.15	25%	64%
1.5	0.15	30%	81%
1.5	0.15	35%	92%
1.5	0.15	40%	98%
1.5	0.15	45%	100%
1.5	0.15	50%	100%
1.5	0.15	55%	100%
1.5	0.15	60%	100%
1.5	0.15	65%	100%
1.5	0.20	25%	48%
1.5	0.20	30%	65%
1.5	0.20	35%	80%
1.5	0.20	40%	91%
1.5	0.20	45%	96%
1.5	0.20	50%	99%
1.5	0.20	55%	100%
1.5	0.20	60%	100%
1.5	0.20	65%	100%

**Table 4:** **Power for comparison of HIV incidence between Arms A and B, with 7 communities per arm and *Population Cohort *** **of 2,500 adults per community (assuming that on average 2,125 (85%) will be HIV-uninfected at baseline and that loss to follow-up will be 20% after 2 years and 25% after 3 years)**

**HIV incidence rate/100 py (control arm)**	**Between-cluster coefficient of variation (k)**	**Effectiveness (%) Arm A**	**Effectiveness (%) Arm B**	**Power (%)**
1.0	0.15	50%	20%	89%
1.0	0.15	50%	25%	78%
1.0	0.15	55%	25%	92%
1.0	0.15	55%	30%	82%
1.0	0.15	60%	25%	98%
1.0	0.15	60%	30%	94%
1.0	0.15	65%	25%	99%
1.0	0.15	65%	30%	99%
1.0	0.20	50%	20%	78%
1.0	0.20	50%	25%	65%
1.0	0.20	55%	25%	83%
1.0	0.20	55%	30%	71%
1.0	0.20	60%	25%	93%
1.0	0.20	60%	30%	87%
1.0	0.20	65%	25%	98%
1.0	0.20	65%	30%	96%
1.5	0.15	50%	20%	94%
1.5	0.15	50%	25%	86%
1.5	0.15	55%	25%	96%
1.5	0.15	55%	30%	90%
1.5	0.15	60%	25%	99%
1.5	0.15	60%	30%	98%
1.0	0.20	65%	25%	99%
1.0	0.20	65%	30%	99%
1.5	0.20	50%	20%	84%
1.5	0.20	50%	25%	72%
1.5	0.20	55%	25%	88%
1.5	0.20	55%	30%	78%
1.5	0.20	60%	25%	96%
1.5	0.20	60%	30%	92%
1.0	0.20	65%	25%	99%
1.0	0.20	65%	30%	98%

Although there is considerable variation in the population size of the 21 study communities (Table 1), a constant sample size was used for the *Population Cohort* in each community to maximize statistical efficiency [67]. A sample of 2,500 adults will ensure that the *Population Cohort* never exceeds 25% of the total adult population. Restricting the size of the cohort relative to the total population helps to minimise the Hawthorne effect of following the cohort on HIV transmission in the wider community.

Sample size calculations for secondary outcomes are presented in the full study protocol [55].

### Ethical considerations

#### Risks and benefits

The mathematical modelling projections indicate that, if the interventions can be implemented as planned with high levels of coverage, there should be a substantial reduction in HIV incidence in Arms A and B compared with Arm C. The results of the HPTN 052 trial and other studies also suggest that there will be some clinical benefit for HIV-positive individuals who commence ART at higher CD4 counts, although clinical effects in those with CD4 counts above 550 cells/μL have not yet been established. Concomitant effects on TB and mother-to-child transmission of HIV are also expected and there may be a reduction in HIV-related stigma as a result of the universal approach to HIV control intrinsic to the PopART interventions.

However, some adverse effects may also be anticipated and are summarised above in the section on ‘Why trials of UTT are needed’. The wide range of potential risks and benefits mandates a rigorously conducted trial to weigh up these effects and to determine whether the effectiveness and cost-effectiveness of the interventions would merit wider scale implementation.

#### Informed consent

The main aspect of the intervention that goes beyond current guidelines is the offer of immediate commencement of ART regardless of CD4 count or clinical stage in Arm A. Written informed consent will be obtained from patients in this arm who are offered immediate treatment that is not considered standard of care according to prevailing national treatment guidelines. Any patients declining this offer will be provided with follow-up and treatment in the same health facilities according to current standard of care.

Written informed consent will be obtained before enrolling individuals in the Population Cohort and case–control studies. Written informed consent will also be required of individuals participating in qualitative research activities that involve collection of participant-identified responses to interviewer questions (such as interviews and focus groups).

The activities of the CHiP team are poised between an established public health intervention (home-based testing and outreach) and a public health research project (data collection and additional follow-up). Household members will be asked to give their verbal consent for participation in this community intervention, which will also permit data collected by CHiPs to be used in aggregate form for research purposes. Individuals will be asked to provide written informed consent for HIV testing as is done routinely at health facilities. In addition, a written information leaflet will be provided to all households.

Full ethical review of the trial protocol has been carried out by the ethics committees of the University of Zambia, Stellenbosch University, London School of Hygiene and Tropical Medicine and the US Centers for Disease Control and Prevention.

## Discussion

Both South Africa and Zambia have seen a recent decrease in the incidence of new HIV infections. However, HIV incidence remains at very high levels and, while it is encouraging that ART reduces mortality, prevalence continues to increase as HIV-infected patients survive for longer [56,57]. Unless more intensive prevention measures can be applied, there will be a continuously expanding number of HIV-infected individuals requiring lifelong treatment, and it will be many years before the epidemic is finally brought under effective control and HIV infection is eliminated as a public health problem.

Two approaches to intensive HIV control are now being explored by public health researchers. Combination prevention acknowledges that single interventions are unlikely to be sufficient to reverse the epidemic while packages that combine a range of proven interventions are more likely to be effective [2,3]. UTT is a new paradigm whereby population-wide HIV testing is combined with effective linkage to care and immediate onset of ART with the aim of maintaining the health of HIV-infected individuals and steeply reducing HIV transmission [68]. The PopART intervention, which includes promotion of male circumcision and other proven interventions as well as UTT, combines these two approaches. A further trial in KwaZulu Natal, South Africa, is also seeking to estimate the effect of ART initiated immediately after HIV diagnosis, irrespective of CD4 count, on the incidence of new HIV infections in the general population over a period of 24 months [69]. Alternative approaches are being investigated in other studies. For example, a trial in Botswana will measure the impact of a strategy combining universal testing with initiation of ART on the basis of HIV viral load as well as CD4 count [70].

The HPTN 071 (PopART) trial in Zambia and South Africa will use a rigorous study design to obtain robust measures of the effectiveness and cost-effectiveness of the intervention in reducing HIV incidence. It will also measure effects on a wide range of important secondary outcomes, including a detailed assessment of potential adverse effects.

The trial has a number of important limitations. First, modelling projections show that the impact of the intervention is expected to build up over time, due to the time needed to achieve full coverage and for the indirect “herd protection” effects of a population-wide intervention on HIV transmission to come into play. The trial will only measure effects for up to 36 months and may therefore underestimate longer-term impact. However, the mathematical models fitted to the trial data will provide long-term projections of effectiveness. Second, the trial will measure the impact of the package of interventions rather than individual components. However, the comparison of Arm A with Arm B will provide information on the additional impact of immediate ART over and above the effect of delivering current interventions with high coverage. Also, the mathematical modelling will allow us to explore the projected effects of different combinations of interventions. Third, there may be some contamination effects due to migration, travel, and sexual contacts across community boundaries. We have tried to minimise these effects by choosing large communities with an average population of around 60,000, but this contamination will lead to some dilution of measured impact. Fourth, attrition in the Population Cohort will lead to some selection bias in our estimate of HIV incidence, so every effort will be made to retain and track Population Cohort participants to minimise the effect of selection bias on effect estimates. Finally, measures of intervention uptake in the Population Cohort may be biased by Hawthorne effects due to repeated follow-up of the cohort over 3 years, including the offer of rapid HIV testing and referral to the clinic for HIV care. This is why we will also collect process data on uptake using data collected by CHiP teams and from health facilities. We will also seek funding for a final *Population Cross-Sectional Survey* in which Hawthorne effects can be avoided.

The central target for the annual coverage of the test and treat campaign is 70%, as shown in Table 2. We acknowledge that this is a challenging target and this is one of the reasons why it is important to carry out the trial to see whether it can be achieved in practice. A systematic review and meta-analysis of home-based testing in sub-Saharan Africa has shown that uptake of home-based testing is high with an overall estimate of 83% acceptance of HIV testing among individuals offered a test, rising to 90% or more in several more recent studies [34]. We believe that the intensity of our intervention – with a team of two CHiPs for every 350 households and several attempts to contact individuals at home to offer HIV testing, followed by active referral to care for HIV-infected individuals combined with strengthened ART services at the clinic – should bring the above target within reach.

UTT not only holds promise for prevention of HIV transmission but also provides impetus for treatment scale-up, thereby reducing morbidity and mortality in HIV-infected individuals. If the trial is completed successfully, the results will provide valuable information on the feasibility, acceptability, and impact of this ambitious population-wide intervention strategy which will inform health policy not only in Zambia and South Africa, but in many other countries with generalised HIV epidemics [16,71]. Our hope is that, if the trial results are encouraging, this new paradigm for HIV control may prove the decisive turning point for this destructive and costly epidemic.

## Trial status

Enrolling.

## Abbreviations

ART: Antiretroviral Therapy; CHiPs: Community HIV-care Providers; DALY: Disability-adjusted life-year; HIV: Human immunodeficiency virus; HPTN: HIV Prevention Trials Network; PMTCT: Prevention of mother-to-child transmission of HIV; QALY: Quality-adjusted life-year; RNA: Ribonucleic acid; STI: Sexually transmitted infection; TB: Tuberculosis; UK: United Kingdom; US: United States (of America); UTT: Universal testing and treatment; WHO: World Health Organization; ZAMSTAR: Zambia-South Africa TB and AIDS Reduction Programme.

## Competing interests

The authors declare that they have no competing interests.

## Authors’ contributions

RH is the principal investigator and SF(2), HA, and NB are co- principal investigators. RH wrote the first draft of the paper and with KS(1) co-wrote revisions and the finalversion of the manuscript. RH, HA, NB, KS(1), SF(1), KS(2), PB, SG, AM, DWJ, CF, SV, and SF(2) contributed to the intellectual content and writing of the paper. All listed co-authors and the HPTN 071 (PopART) Study Team contributed to the original design and development of the trial protocol. All listed authors have read and approved the final manuscript.

## HPTN 071 (PopART) Study Team (in addition to named authors)

Yaw Agyei (Department of Pathology, Johns Hopkins University School of Medicine, Baltimore, Maryland, USA), Megan Baldwin (Vaccine and Infectious Disease Division, Fred Hutchinson Cancer Research Center, Seattle, Washington, USA), Mark Barnes (Ropes & Gray LLP, Boston, Massachusetts, USA), Virginia Bond (London School of Hygiene and Tropical Medicine, London, UK and ZAMBART, University of Zambia, School of Medicine, Ridgeway Campus, Lusaka, Zambia), David Burns (Division of AIDS, National Institute of Allergy and Infectious Diseases, US National Institutes of Health, Bethesda, Maryland, USA), Nathaniel Chishinga (ZAMBART, University of Zambia, School of Medicine, Ridgeway Campus, Lusaka, Zambia), Vanessa Cummings (Department of Pathology, Johns Hopkins University School of Medicine, Baltimore, Maryland, USA), Deborah Donnell (Vaccine and Infectious Disease Division, Fred Hutchinson Cancer Research Center, Seattle, Washington, USA), Lynda Emel (Vaccine and Infectious Disease Division, Fred Hutchinson Cancer Research Center, Seattle, Washington, USA), Susan Eshleman (Department of Pathology, Johns Hopkins University School of Medicine, Baltimore, Maryland, USA), Peter Godfrey-Faussett (London School of Hygiene & Tropical Medicine, London UK), Elizabeth Greene (FHI 360, Durham, North Carolina, USA), James Hargreaves (Department of Infectious Disease Epidemiology, London School of Hygiene & Tropical Medicine, London, UK), Katharina Hauck (Business School, Imperial College London, London, UK), Tanette Headen (FHI 360, Durham, North Carolina, USA), Lyn Horn (Desmond Tutu TB Centre, Stellenbosch University, South Africa), Peter Kim (Division of AIDS, National Institute of Allergy and Infectious Diseases, US National Institutes of Health, Bethesda, Maryland, USA), Estelle Piwowar-Manning (Department of Pathology, Johns Hopkins University School of Medicine, Baltimore, Maryland, USA), Katie McCarthy (FHI 360, Durham, North Carolina, USA), Maurice Musheke (ZAMBART, University of Zambia, School of Medicine, Ridgeway Campus, Lusaka, Zambia), Albert Mwango (ZAMBART, University of Zambia, School of Medicine, Ridgeway Campus, Lusaka, Zambia), Alwyn Mwinga (ZAMBART, University of Zambia, School of Medicine, Ridgeway Campus, Lusaka, Zambia), Monde Muyoyeta (ZAMBART, University of Zambia, School of Medicine, Ridgeway Campus, Lusaka, Zambia), Musonda Simwinga (ZAMBART, University of Zambia, School of Medicine, Ridgeway Campus, Lusaka, Zambia), Ab Schaap (ZAMBART, University of Zambia, School of Medicine, Ridgeway Campus, Lusaka, Zambia), Peter C. Smith (Business School, Imperial College London, London, UK), Shauna Wolf (Department of Pathology, Johns Hopkins University School of Medicine, Baltimore, Maryland, USA), and Rhonda White (FHI 360, Durham, North Carolina, USA).
